# Looking at the Ventriloquist: Visual Outcome of Eye Movements Calibrates Sound Localization

**DOI:** 10.1371/journal.pone.0072562

**Published:** 2013-08-29

**Authors:** Daniel S. Pages, Jennifer M. Groh

**Affiliations:** 1 Center for Cognitive Neuroscience, Duke University, Durham, North Carolina, United States of America; 2 Department of Psychology and Neuroscience, Duke University, Durham, North Carolina, United States of America; 3 Department of Neurobiology, Duke University, Durham, North Carolina, United States of America; Istituto di Neuroscienze, Italy

## Abstract

A general problem in learning is how the brain determines what lesson to learn (and what lessons not to learn). For example, sound localization is a behavior that is partially learned with the aid of vision. This process requires correctly matching a visual location to that of a sound. This is an intrinsically circular problem when sound location is itself uncertain and the visual scene is rife with possible visual matches. Here, we develop a simple paradigm using visual guidance of sound localization to gain insight into how the brain confronts this type of circularity. We tested two competing hypotheses. 1: The brain guides sound location learning based on the synchrony or simultaneity of auditory-visual stimuli, potentially involving a Hebbian associative mechanism. 2: The brain uses a ‘guess and check’ heuristic in which visual feedback that is obtained after an eye movement to a sound alters future performance, perhaps by recruiting the brain’s reward-related circuitry. We assessed the effects of exposure to visual stimuli spatially mismatched from sounds on performance of an interleaved auditory-only saccade task. We found that when humans and monkeys were provided the visual stimulus asynchronously with the sound but as feedback to an auditory-guided saccade, they shifted their subsequent auditory-only performance toward the direction of the visual cue by 1.3–1.7 degrees, or 22–28% of the original 6 degree visual-auditory mismatch. In contrast when the visual stimulus was presented synchronously with the sound but extinguished too quickly to provide this feedback, there was little change in subsequent auditory-only performance. Our results suggest that the outcome of our own actions is vital to localizing sounds correctly. Contrary to previous expectations, visual calibration of auditory space does not appear to require visual-auditory associations based on synchrony/simultaneity.

## Introduction

Sound location is inferred from direction-dependent differences in sound timing, loudness, or frequency content at the two ears [1], [2]. The spatial location associated with a given value of any of these cues varies with head size, the immediate acoustic environment, and the spectral content of the sound itself [3], [4]. The brain appears to solve this problem in part by using vision to calibrate its estimate of sound location [5], [6]. Studies of ventriloquism demonstrate an immediate visual capture of the perceived location of sounds when paired with visual stimuli at a different location in space [7]–[13], and studies of prism adaptation and the ventriloquism aftereffect show that such a shift can persist in the absence of the visual stimulus [14]–[20].

How the brain uses vision to accomplish this calibration is unclear. Since there are always many objects in view, it is puzzling how we know which visual object corresponds to a sound, especially when we cannot be sure exactly where the sound is coming from. Our study considered two specific possibilities. First, the brain might use temporal synchrony between sights and sounds to determine the location of a sound based on vision. That is, a visual stimulus that occurs at the same time as a sound, such as the sight of a ball hitting a bat, might be assumed to be the source of the accompanying cracking sound. It has long been known that timing is critical for the ventriloquism effect [7], [10], [21], [22], but that does not necessarily mean that this same mechanism explains the lasting changes observed in prism adaptation and in the ventriloquism aftereffect. Temporal synchrony would be expected to be most powerful for dynamic stimuli (those showing changes over time such as onset/offset or motion), in which individual temporal variation provides a basis for assessing coincidence, although simple simultaneity could also play a role for static, continuously present stimuli that overlap in time.

An alternative involves visual feedback: what you see after you make an eye movement to a sound could guide learning in sound localization. According to this view, the brain might move the eyes to view the estimated location of a sound in order to determine whether the sound location was perceived correctly. For example, if when you look towards where you think you heard the ball hitting the bat, you see a batter dropping his bat and running for first base, you have perceived the sound’s location correctly. If you see a pitcher looking mournful, you have missed. Next time you hear that cracking sound, you will adjust your estimate of sound location in favor of the direction of home plate and away from the pitcher’s mound. Again, such a mechanism could apply both to stimuli that are dynamic and to those that are static or continuously present, the only requirement being the persistence of the visual stimulus long enough to provide feedback following an eye movement initiated by the sound.

In the real world, these two potential mechanisms often overlap: many visual and auditory stimuli occur synchronously and are present long enough that visual feedback after completing an eye movement can be obtained. In an experimental setting, these two mechanisms can be dissociated from each other by adjusting the timing of the visual stimulus with respect to the sound: if the visual stimulus occurs temporally coincident with a brief sound prior to an eye movement, then only temporal synchrony cues are available. In contrast, if the visual stimulus is presented asynchronously with the brief sound, but is timed to be present immediately after the eye movement, then only visual feedback is available.

## Results

**Figure 1:**
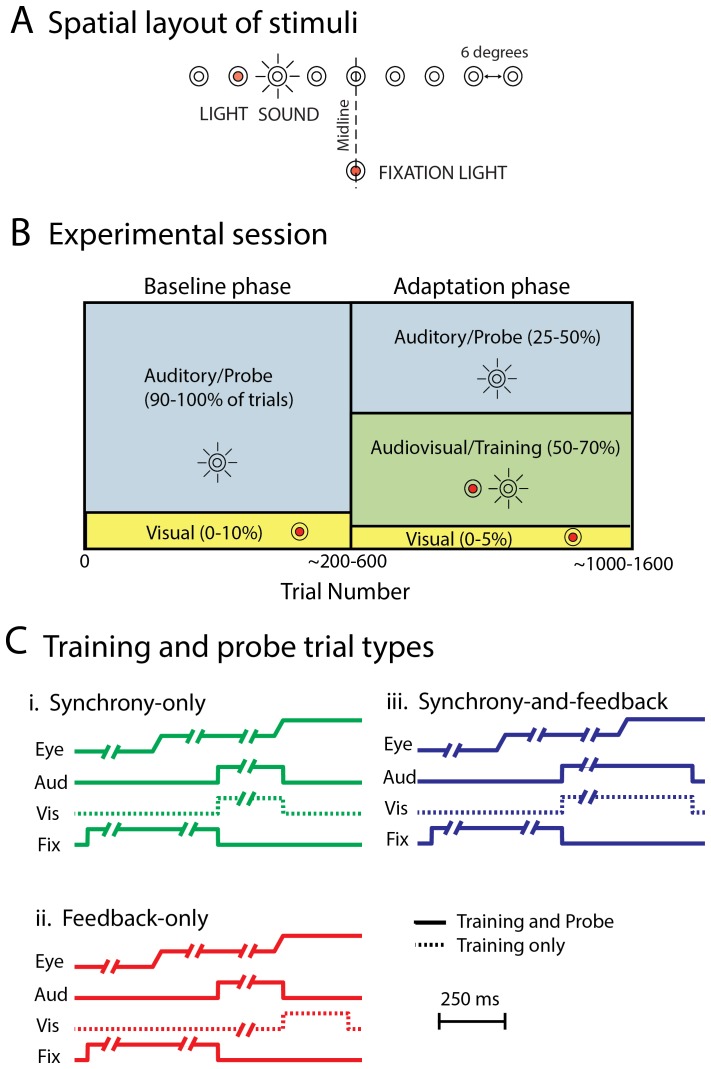
Spatial Configuration of Stimuli, Experimental Session Design, and Trial Types. (A) All trials began with fixation of a visual fixation target located 12 degrees below a row of speakers and LEDs spanning −24 to 24 degrees along the horizontal meridian. Spatially mismatched visual-auditory trials involved the presentation of a sound at one speaker combined with the illumination of an LED attached to the adjacent speaker. (B) Each experimental session involved an initial baseline assessment of performance on auditory and visual trials followed by an adaptation phase in which training trials with mismatched audiovisual stimuli were interleaved in proportions as illustrated. Performance on auditory probe trials after introduction of these mismatched audiovisual training trials was compared to baseline performance. (C) Three types of audiovisual training and auditory probe trials were assessed in different sessions. On *synchrony-only* audiovisual training trials, the visual and auditory stimuli were coincident in time but both were turned off during the subject’s saccade to the target, preventing visual feedback. On *feedback-only* audiovisual training trials, the sound came on first, but it was turned off and a visual stimulus was turned on during the saccade to the sound, eliminating synchrony between the visual and auditory stimulus. On *feedback-and-synchrony* audiovisual trials, the auditory and the visual stimulus were turned on together, and were left on following completion of the saccade, thus allowing a synchronous visual stimulus to also provide visual feedback. Interleaved auditory-only probe trials differed from the corresponding audiovisual training trials in lacking a visual stimulus but were otherwise identical.

We tested these theories in humans and rhesus monkeys (*Macaca mulatta*) using audiovisual **training** tasks designed to dissociate these two potential mechanisms (**figure 1C**). Participants were required to make an eye movement from a fixation LED to a sound under different auditory and visual conditions. (**Visual-only** trials were included for calibration purposes.) In the **synchrony-only** task, a visual stimulus appeared at the same time as a sound but too briefly to provide feedback after an eye movement to that sound. In the **feedback-only** task, the visual stimulus appeared during the execution of an eye movement to a sound, but was never on at the same time as the sound. A third task (“**synchrony-and-feedback**”) involved visual and auditory stimuli presented synchronously and for a sufficient time to provide feedback following the saccade. This task was included for comparison with previous studies of prism adaptation and the ventriloquism aftereffect in which both synchrony and feedback cues were likely to be available [6], [14], [21], [23], [24]. In all audiovisual **training** trials, the visual stimuli were mismatched in space by 6 degrees to the left or right of the sounds (**figure 1A**), with a consistent shift direction for each session.

We assessed the effects of these audiovisual **training** trials on the endpoints of eye movements on interleaved auditory **probe** trials which were identical to the audiovisual trials except that only the sounds were presented. We compared performance on **probe** trials before and after the introduction of the audiovisual **training** trials (**figure 1B**).

**Figure 2:**
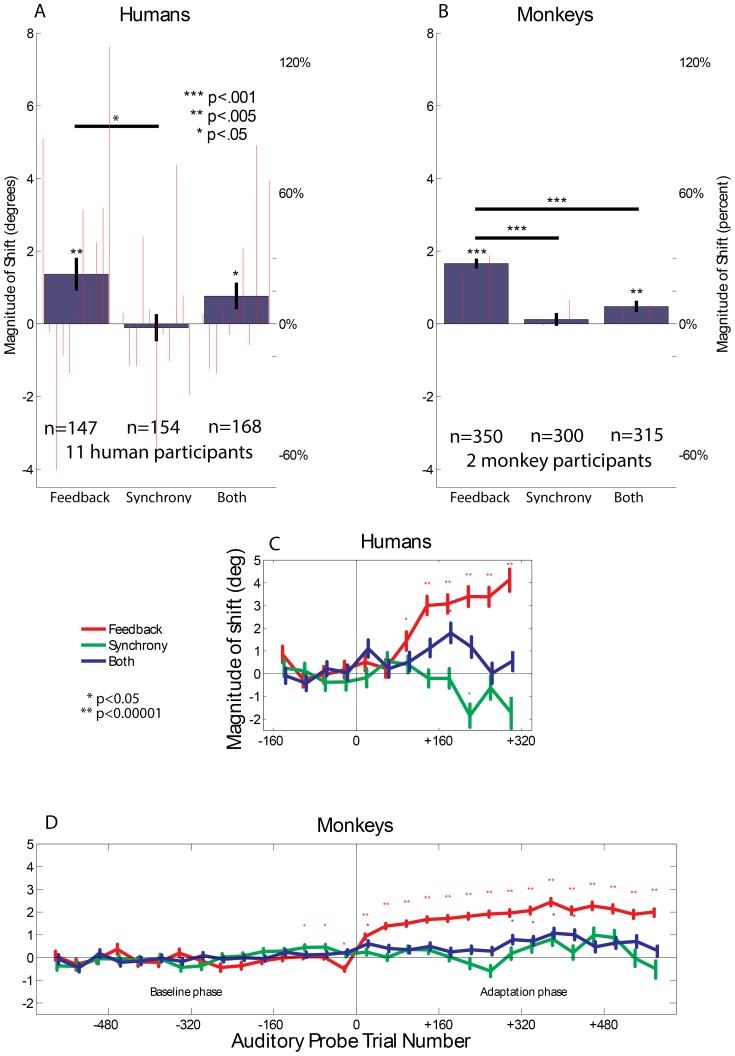
Robust induced shifts in Feedback condition. The mean change +/− SE in saccade endpoints on auditory probe trials during the adaptation phase compared to the baseline phase for humans (A) and monkeys (B). The data is normalized such that the direction of the displaced visual stimulus is always considered positive. Small red bars represent individual subject totals, with the human subjects ordered by their performance on the baseline trials (best auditory saccade precision on the left) N values correspond to speaker positions*sessions (see Materials and Methods). (C, D) The time course of the shift in behavioral performance as a function of auditory-probe trial number with respect to the beginning of the adaptation phase (mean +/− SE). For example, trials 120–160 represent the 120^th^–160^th^ auditory probe trial after audiovisual trials began. Each auditory probe trial is considered a data point. The data in figure 2C–2D is jittered on the X axis for readability.

We found that the feedback-only task exerted a much more powerful effect on the estimation of sound location than the **synchrony-only** task did: on average, subjects altered their sound-locating eye movements in the direction of the lights’ location to a greater degree when the visual stimulus was presented as feedback than when it was presented at the same time as the sound (**figure 2A–B**, analysis of variance, significant interaction between task type and baseline vs. adaptation phase, p<0.0001). In the **feedback-only** task, a six-degree separation between the visual and auditory stimuli produced an induced shift averaging 1.3 degrees (21%) in humans and 1.7 degrees (28%) in monkeys (post-hoc Student’s t-test, p<0.005 for humans and p<0.0001 for monkeys).

Particularly in monkeys, this occurred quite rapidly: the shift was statistically significant within the first 40 trial bin of auditory **probe** trials after the introduction of **training** trials (p<0.0001, bonferroni corrected for the number of time bins tested), and had reached a shift value of approximately 1.2 degrees or 20% by the second 40-trial bin (**figure 2C–D**, red traces). By contrast, the behavioral shifts induced in the **synchrony-only** task were not significantly different from zero overall (**figure 2A–B**). Note that this analysis considers each trial to be a data point, thus increasing the total N and therefore the sensitivity of the test. However, even in this less conservative case the synchrony condition caused no individual time bins with a significant shift (p<0.05) in the predicted direction (**figure 2C–D**, green traces). Though the effect briefly became weakly significant in the *opposite* direction of what would be expected under ventriloquism aftereffect, this could be a result the less conservative nature of the analysis and in any case is not consistent with synchrony eliciting a shift in the direction of the visual stimulus under the conditions of our paradigm.

**Feedback-only** also induced a larger shift in reported sound location than the **synchrony-and-feedback** task, although the latter task did produce a significant shift in auditory saccade endpoints toward the visual stimulus (**figure 2A–B**). This shift was statistically significant within some time bins but not others during the adaptation phase (**figure 2C–D**, blue traces, some p’s<0.05, bonferroni corrected). The smaller observed effect on the **synchrony-and-feedback** task compared to the **feedback-only** task suggests that synchrony or simultaneity may actually serve to diminish the impact of visual stimuli on sound calibration, perhaps because the sound is redundant when the visual stimulus is already present. Subjects may simply localize the visual stimulus and ignore the sound, and the learning process may therefore not be as actively engaged.

Intersubject variability was modest in monkeys but greater in human subjects, as illustrated by the red lines in the bar graph in **figure 2A–B**. This variability was related to baseline skill at performing sound localization tasks. The individual subject bars are ordered by decreasing baseline accuracy of performing auditory-only saccades (correlation coefficient of saccade endpoint with target location ranging from 0.94 to 0.43 in the humans and 0.81 to 0.76 in the monkeys). The human subjects who demonstrated less precision in their auditory saccade accuracy initially were more influenced by the visual stimuli in the feedback condition (red bars in feedback case of **figure 2A** increase in height; linear regression of induced shift during feedback vs. correlation coefficient of relationship between auditory saccade endpoint and target location during baseline, p<0.05).

This pattern of individual differences is consistent with previous studies showing that subjects weigh stimuli more heavily when they are considered more reliable, and that this weighting produces a statistically optimal estimate of location [13], [25]. We investigated this possibility further using subjects’ responses on the audiovisual training trials during the synchrony and synchrony-and-feedback sessions in which visual and auditory stimuli were presented contemporaneously. We calculated the predicted relative weights of the visual (wVis_predicted_) and auditory (wAud_predicted_) stimuli under an optimal cue combination model, using the mean of the standard deviations to each speaker position per session (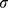
Vis and 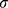
Aud for visual and auditory saccades, respectively) as follows.
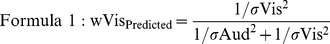


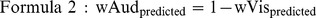



We then calculated the ‘empirical’ visual (wVis_empirical_) and auditory (wAud_empirical_) weightings by comparing the actual saccade endpoints of baseline visual (Vis_sac_) and auditory (Aud_sac_) saccades to the endpoints of bimodal saccades (Bi_sac_).
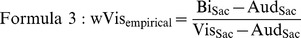


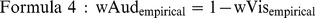



The predicted visual weightings under an optimal cue combination model were wVis_predicted_ = 0.8383, and wVis_predicted_ = 0.9372 for monkeys and humans respectively, while the empirically observed visual weightings were only wVis_empirical = _0.6491 and wVis_empirical = _0.3779 for monkeys and humans respectively. This result suggests that our subjects were weighting the auditory stimulus more heavily than would be predicted under an optimal cue combination model. This is probably because our task instructions explicitly (humans) or implicitly (monkeys, through centering of reward windows) directed subjects to localize the auditory stimulus. Prior work where human subjects are instructed to locate the sound and not the visual distractor have found amounts of visual capture broadly consistent with ours [17], [26]. Had we used the method of instructing the subjects to localize a single fused percept [13], we would expect to observe near-optimal cue combination in the bimodal trials in the **synchrony** and **synchrony-and-feedback** trial types. Our results are consistent with the hypothesis that the relative weightings of visual and auditory stimuli in a bimodal localization task can be near-optimal if the subjects are instructed to seek a bimodal stimulus, auditory-weighted if the subjects are instructed to seek the auditory stimulus, or visual-weighted if the subjects are instructed to seek the visual stimulus.

**Figure 3:**
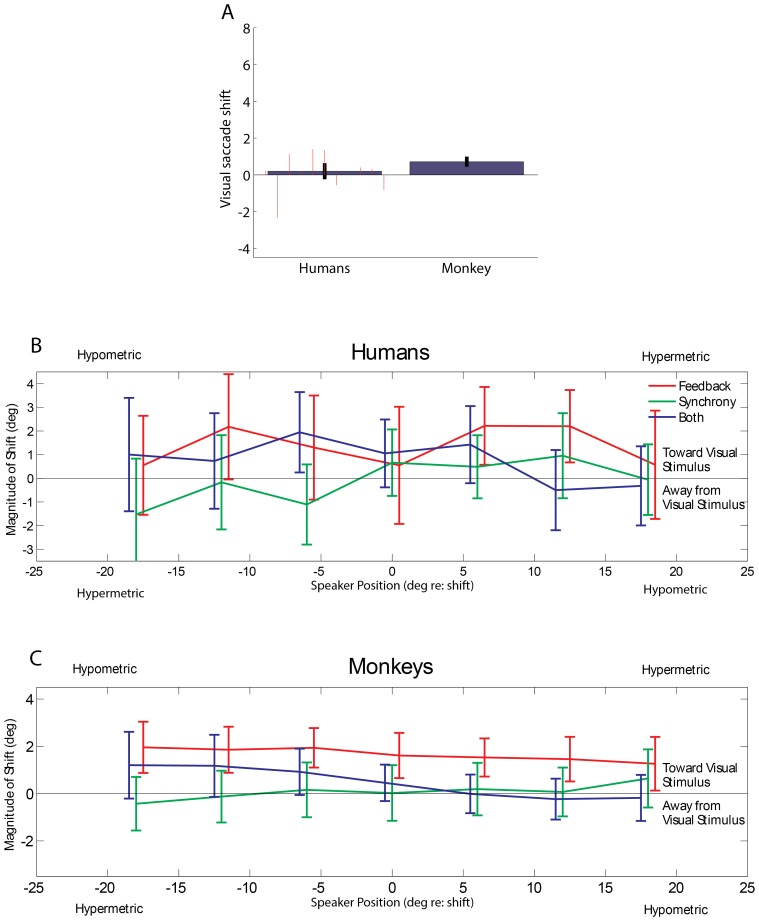
Visual control and auditory shift by speaker position. (A) Shift on visual trials. The magnitude of the induced visual shift on visual-only trials during feedback sessions was calculated in the same fashion as for auditory-only trials; that is, by subtracting the mean baseline visual saccade amplitude for a given target location from that of the adaptation phase, yielding one value per session and target. The bars represent the means of these values +/− the standard error (same conventions as figure 2). (B–C). Shift on auditory trials by speaker position. Magnitude of shift is plotted as a function of speaker location for humans (3B) and monkeys (3C). The Y axis is normalized such that the direction of the visual stimulus’ displacement is always plotted as positive. The X axis normalized such that a shift in the direction of the visual stimulus involves hypermetric (longer) saccades for positive X values and hypometric (shorter) saccades for negative X values. The feedback condition elicited a larger shift in the direction of the visual stimulus than the simultaneous condition did, regardless of whether the saccades involved were made longer or shorter.

Finally, we considered whether the plasticity evoked by the visual trials was likely to occur within the auditory pathway or within the oculomotor pathway, or some combination of the two. If changes occur within the motor pathway, they should generalize to saccades evoked by other target modalities. In one monkey (monkey N) and the humans (**figure 3A**), we tested whether there was any evidence of a shift in performance on the visual trials interleaved with the feedback training trials. The shift observed in humans was small and not significant (0.20 degrees). In monkey N, the shift was also smaller than that observed on auditory trials, 0.71 degrees, but was significantly different from zero (p<0.05, Student’s T test), suggesting that a modest portion of the plasticity may be implemented in parts of the pathway shared in common between visual and auditory stimuli. However, a second tendency that has been observed in previous paradigms involving saccade adaptation [27], [28] was not observed here. In such studies, the induced shift tends to be larger when it involves shortening a saccade rather than lengthening it [29]. We did not see such an effect: the size of the induced shift on auditory trials during feedback sessions was consistent across different target locations regardless of whether the shift involved shortening or lengthening saccades (**figure 3B–C**). Taken together, these findings suggest that although a portion of the plasticity may occur within the oculomotor pathway, the remainder is likely to occur within the auditory pathway. One possible future experiment to further test this hypothesis is to assess whether the ventriloquism aftereffect will generalize to other types of sound localization behaviors using different motor effectors.

## Discussion

Here, we considered several precisely controlled timing paradigms that limited when visual information is available to provide guidance over sound location perception, and we identified visual feedback provided at the end of an eye movement as a particularly important factor for recalibrating auditory space. In everyday sensory environments, the relative timing of visual and auditory information occurs on a continuum, and cognitive knowledge of the common causes of visual and auditory stimuli likely plays a role. For example, if you see a refrigerator and a tree and hear a continuous humming noise, you may assume the noise comes from the refrigerator rather than the tree. Although these stimuli are simultaneous, this discrimination cannot be a result of that simultaneity because temporal cues offer no ability to discriminate between the refrigerator and the tree. Instead you may make the determination based on a cognitive judgment about what kinds of noise ‘belongs’ with refrigerators. These cognitive ‘belongingness’ cues were likely minimal for the LEDs and broadband noise stimuli in our study, and in any case they were held constant across the different conditions of our study so they could not be responsible for any observed differences. However, such cognitive cues may well exert their effects in concert with eye-movement related visual feedback: recognizing candidate visual sources of sounds will often require foveal vision (particularly for smaller visual stimuli such as a beeping cell phone or the mouth of a person speaking).

The neural mechanisms that allow visual feedback to recalibrate auditory space are uncertain. Converging lines of evidence suggest that visual signals are present in several auditory areas of the brain [30]–[33]. These signals may provide information about the accuracy of (or error in) attempts to localize sounds. Alternatively, visual areas elsewhere may produce a signal that indicates success or failure in foveating a target. This visual success/failure signal could then be transmitted via reward-related circuitry, which is known to influence the auditory pathway [34], [35].

Our results share similarities with two related phenomena. As noted above, when a visual stimulus jumps to a new position during the course of an eye movement, subjects gradually learn to anticipate this jump and make saccades shifted toward the future location of the visual stimulus, a phenomenon known as saccade adaptation [27], [28], [36], [37]. The feedback-only paradigm used here can be thought of as a cross-modal version of this saccade adaptation task. The use of feedback occurring at one point in time to alter neural circuitry based on patterns of activity that occurred earlier suggests that visual calibration of auditory space may be thought of as a type of prediction error. As such, the underlying mechanism is not exclusively driven by mutual strengthening of co-activated visual and auditory circuits, as originally envisioned by Hebb as a mechanism of associative learning [38], [39]. Instead, like prediction error or reinforcement learning, visual feedback may function as a consequence signal, e.g. a form of reward when performance is successful.

While we failed to find a statistically significant shift of auditory perception in the direction of the visual stimulus under the synchrony condition, we do *not* make the strong claim that there is exhaustively no circumstance under which temporal synchrony can contribute to the ventriloquism aftereffect. Instead, our key findings are (1) that feedback can be a major driver of the ventriloquism aftereffect, and (2) under the conditions of our experiment, feedback induces a stronger shift than temporal synchrony.

Ventriloquists take pains to provide a synchronous visual stimulus – the puppet’s mouth moving in time with the puppeteer’s speech – at the location they would like you to perceive the voice. Why do this if synchrony is not important to the persistent changes involved in visual calibration of auditory space? Enhancing the cognitive ‘belongingness’ as described above is one possibility - when stimuli are dynamic, they should be synchronous in order to match cognitive expectation and produce the greatest verisimilitude.

A second possibility is that the mechanisms that guide plasticity may be distinct from those that influence perception in the moment. In other words, despite the similar terminology, the ventriloquism *aftereffect* may involve separate mechanisms from those involved in the ventriloquism *effect*. Synchrony may be more important for the immediate perception of the ventriloquism effect [7], [10], [21], [22], but our results suggest that feedback is the more powerful driver of the ventriloquism aftereffect. Future work is needed to investigate how feedback and synchrony-dependent mechanisms might work in concert with the learned associations between auditory and visual stimuli that lead us to expect to hear speech sounds emanating from moving mouths.

## Materials and Methods

### Ethics Statement

Eleven humans (six male, ages 18–55) and two rhesus monkeys (*Macacca mulatta*, one male, ages 4–9) participated in the studies. The human and animal experimental protocols were approved by the Institutional Review Board and the Institutional Animal Care and Use Committee of Duke University, respectively. All human participants provided written informed consent prior to participating in the study. Animal procedures were conducted in accordance with the principles of laboratory animal care of the National Institutes of Health (publication 86–23, revised 1996) and involved standard operative and post-operative care including the use of anesthetics and analgesics for all surgical procedures.

Specifically, prior to surgery, animals were pre-anesthetized with ketamine (I.M., 5–20 mg/kg) or ketamine/dexdomator (I.M., 3.0 mg/kg ketamine and 0.075–0.15 mg/kg dexdomitor) and were maintained under general anesthesia with isoflourine (inhalant, 0.5–3.0%). Systemic anti-inflammatory medications (dexamethasone, flunixin, or keterolac) were given as indicated by veterinarian. After surgery, animals were treated with burprenorphine analgesic (I.M., 0.01–0.02 mg/kg doses) for three days.

Animals were housed in a standard macaque colony room in accordance with NIH guidelines on the care and use of animals. Specifically, the animals were housed in Primate Products Apartment Modules (91 cm*104 cm*91 cm), including pair or group housing when compatible partner monkeys were available. Pair and group housed animals exhibited species-typical prosocial behavior such as grooming. Animals also had frequent access to Primate Products Activity Modules (91 cm*104 cm*183 cm), allowing for more exercise including a greater range of vertical motion. All animals had visual and auditory interactions with conspecifics in the room (∼10 animals). Animals were enriched in accordance with the recommendations of the USDA Environmental Enhancement for Nonhuman Primates (1999), and the National Research Council’s Psychological Well-Being of Nonhuman Primates (1998), and the enrichment protocol was approved by the IACUC. Specifically, the animals had access to a variety of toys and puzzles (eg Bioserv dumbbells (K3223), Kong toys (K1000), Monkey Shine Mirrors (K3150), Otto Environmental foraging balls (model 300400) and numerous other toys and puzzles). Material from plants such as Bamboo and Manzanita was also placed in the cage to give the animals additional things to climb on and interact with. The temperature in the animal facilities was 20–25 degrees C and the colony room was kept on a 12 hr/12 hr light/dark cycle. The animals had approximately an hour of audiovisual contact with at least two (and often several) humans per day. The animals’ diets consisted of monkey food (LabDiet 5038 or LabDiet 5045) delivered twice a day, plus daily supplementary foods such as bananas, carrots, mango, pecan nuts, dried prunes, or other treats (typically acquired from local supermarkets or online vendors) to add variety to the animals’ diets. No animals were sacrificed during this study - at the time of the submission of this manuscript both animals that participated in this study are in good general health.

### Apparatus and General Procedures

Apparatus and general procedures are described in detail elsewhere [17], [40]. Briefly, experiments were conducted in a darkened, single walled sound attenuation chamber (Industrial Acoustics) and lasted roughly 2–3 hours including setup. Participants’ heads were restrained to minimize movements via head post (Crist instruments) for monkeys, or chin rests for humans. For humans and animal N, eye position was measured with an infrared video eye tracking system (Eyelink). For animal F, eye position was measured using a scleral search coil [41], [42]. In either case, the eye tracking system was calibrated prior to data collection using a standard five-point procedure.

### Stimuli

Stimulus delivery and data acquisition was controlled by a personal computer running Beethoven (Ryklin Software). The auditory stimuli consisted of broadband noise (each sample independently randomly generated) calibrated to 55 (+/−1) db SPL, delivered through speakers (Cambridge Soundworks) approximately 120 cm in front of the participant and ranging from 18 degrees left from straight ahead to 18 degrees right from straight ahead in 6 degree intervals. Visual stimuli consisted of LEDs attached to the faces of the speakers. Mismatched visual and auditory stimuli were presented by delivering a sound through one speaker and a visual stimulus via the adjacent LED, i.e. 6 degrees apart.

### Behavior

Monkeys were trained by operant conditioning to saccade to visual and auditory stimuli for a liquid reward. When spatially-mismatched visual and auditory stimuli were presented, the animals’ reward window was centered on the auditory stimulus but large enough to include both (16–24 degrees in diameter). The large response windows ensured the animals were not explicitly forced to choose between the visual or auditory stimuli as the target of the saccade. Humans were instructed to look at the sound if it was present, or to look at the light if there was only a light. Because saccade endpoint was used as a dependent measure, all trials were included for analysis as long as there was a saccade, regardless of where the subjects looked.

The behavioral tasks are illustrated in Figure1C. The timing of several phases of the trial was variable either due to the subjects’ behavior or under experimental control. On trials involving stimuli turning or remaining on during/following the saccade (**Feedback-only** and **Synchrony-and-Feedback**), these stimuli remained on for an additional 250 ms.

### Data Analysis

Saccades were detected using a velocity based algorithm (EyeMove software). All other data analysis was done in MATLAB (Mathworks). ‘Magnitude of induced shift’ was the horizontal difference between auditory saccades during the baseline phase and auditory saccades during the adaptation phase. The mean value of the induced shift for a given speaker position and session was considered a data point for the statistical tests, except in the longitudinal analysis depicted in figures 2C and 2D where each saccade to an auditory probe was considered a data point.
